# Spinal Cord Injury Increases Pro-inflammatory Cytokine Expression in Kidney at Acute and Sub-chronic Stages

**DOI:** 10.1007/s10753-021-01507-x

**Published:** 2021-08-21

**Authors:** Shangrila Parvin, Clintoria R. Williams, Simone A. Jarrett, Sandra M. Garraway

**Affiliations:** 1 Department of Physiology, Emory University School of Medicine, 615 Michael Street, Suite 605G, Atlanta, GA 30322 USA; 2 Neuroscience, Cell Biology and Physiology, Wright State University, Dayton, OH USA; 3 Department of Physiology, Emory University School of Medicine, 615 Michael Street, Suite 605G, Atlanta, GA 30322 USA

**Keywords:** cytokines, c-fos, systemic inflammation, kidney, spinal cord injury.

## Abstract

Accumulating evidence supports that spinal cord injury (SCI) produces robust inflammatory plasticity. We previously showed that the pro-inflammatory cytokine tumor necrosis factor (TNF)α is increased in the spinal cord after SCI. SCI also induces a systemic inflammatory response that can impact peripheral organ functions. The kidney plays an important role in maintaining cardiovascular health. However, SCI-induced inflammatory response in the kidney and the subsequent effect on renal function have not been well characterized. This study investigated the impact of high and low thoracic (T) SCI on C-fos, TNFα, interleukin (IL)-1β, and IL-6 expression in the kidney at acute and sub-chronic timepoints. Adult C57BL/6 mice received a moderate contusion SCI or sham procedures at T4 or T10. Uninjured mice served as naïve controls. mRNA levels of the proinflammatory cytokines IL-1β, IL-6, TNFα, and C-fos, and TNFα and C-fos protein expression were assessed in the kidney and spinal cord 1 day and 14 days post-injury. The mRNA levels of all targets were robustly increased in the kidney and spinal cord, 1 day after both injuries. Whereas IL-6 and TNFα remained elevated in the spinal cord at 14 days after SCI, C-fos, IL-6, and TNFα levels were sustained in the kidney only after T10 SCI. TNFα protein was significantly upregulated in the kidney 1 day after both T4 and T10 SCI. Overall, these results clearly demonstrate that SCI induces robust systemic inflammation that extends to the kidney. Hence, the presence of renal inflammation can substantially impact renal pathophysiology and function after SCI.

**Supplementary Information:**

The online version contains supplementary material available at 10.1007/s10753-021-01507-x.

## INTRODUCTION

Spinal cord injury (SCI) causes many devastating health challenges during the acute and chronic stages post-injury. Renal complication resulting in renal failure can contribute to mortality after SCI. In fact, whereas renal failure may no longer represent the leading cause of death in SCI patients, renal dysfunction following SCI is well documented [1–3]. Also, because the kidney is a main regulator of blood pressure, compromised renal function after SCI can indirectly contribute to blood pressure dysregulation, and consequently, impaired cardiovascular health, a leading cause of morbidity and mortality for spinal cord injured patients [4–6]. A hallmark feature of SCI is the initiation of an inflammatory response typically at or near the lesion epicenter [7]. These events can be expressed as increases in glial cell migration and proliferation response, release of inflammatory cytokines, and subsequent apoptotic cascades. For example, we have previously shown an increase in the pro-inflammatory cytokine tumor necrosis factor-alpha (TNFα) and several of its downstream signaling molecules in the lesioned spinal cord after SCI [8, 9].

Recent reports have shown that SCI causes a systemic inflammatory response that can affect peripheral organ function [10, 11]. Immune and inflammatory cells phenotypes [10, 12] and pro-inflammatory cytokines such as TNFα, interleukin (IL)-1β, and IL-6 [13] are increased in peripheral tissue, including the kidney, after trauma. Like the inflammatory cytokines, the proto-oncogene C-fos, is significantly upregulated in the spinal cord after SCI and is similarly increased in the kidney in diseased or injured conditions [14–17]. Thus, the kidney, like other organ systems, is susceptible to the systemic inflammatory response after SCI [10, 11].

In human and animal studies, hypertension is associated with inflammation [18, 19], which suggests that inflammatory mediators can promote renal pathophysiology, and possibly cardiovascular dysfunction after SCI. However, a thorough investigation of the expression profiles of key pro-inflammatory cytokines in the kidney as a first-step interrogation into the effect SCI has on kidney pathophysiology and renal dysfunction has not been undertaken. In this study, we investigated whether SCI induces a systemic inflammatory response, extending to the kidney, that is independent of the injury level. To accomplish this, we utilized a commonly used contusion SCI at two distinct spinal levels [thoracic (T) 4 and T10]. SCI at T4 is often used to examine cardiovascular dysfunction, including autonomic dysreflexia after SCI, and related plasticity (e.g., [20, 21]). Meanwhile, injury at T10 is frequently employed to assess behavioral deficits such as impaired locomotor [22, 23] and bladder function [24], and the development of neuropathic pain [25–27]. Because SCIs at both high and low thoracic levels have been shown to impact behavioral outcomes and importantly, induce secondary cellular plasticity (e.g., [28–30]), the current study examines the expression profiles of C-fos, TNFα, IL-1β, and IL-6 in the kidney of adult mice after contusion SCI at T4 and T10. These cellular assessments were undertaken at an acute and a sub-chronic timepoint after SCI. In addition, we profiled changes in the expression levels of these inflammatory mediators in the lesioned spinal cord.

## METHODS

### Animals

Experiments were performed in female and male wild-type C57BL/6 mice which were bred in our animal colony or purchased from Jackson Laboratory. Mice were approximately 3–4 months old at the time of surgery and weighed 20–22 g (females) and 24–26 g (males). They were housed in standard cages in a vivarium on a 12:12-h light–dark cycle and fed standard rodent diets ad libitum. Experimental procedures were approved by the Animal Care and Use Committee of Emory University and conformed to national standards for the care and use of experimental animals and the American Physiological Society’s “Guiding Principles in the Care and Use of Animals.”

### SCI Surgical Procedure

Mice were deeply anesthetized with isoflurane (5%, gas; lowered to 2–3% once stable anesthesia was achieved). Under sterile conditions, a skin incision and dorsal laminectomy exposed the underlying spinal cord at T4 or T10. For midline contusion injuries, mice received a 70 kdyne, zero dwell time, impact onto the dorsal surface of the spinal cord with an Infinite Horizon impactor (Precision Systems and Instrumentation, Fairfax Station, VA). Care was taken to ensure that dorsal roots were not damaged by the laminectomy or impact, and on-target bilateral bruising of the dorsal spinal cord was verified by examination under a dissecting microscope. The overlying muscle and skin were sutured and the wound area treated with triple antibiotic ointment (bacitracin-neomycin-polymyxin B) topically. Sham control mice underwent the same surgical procedure but without receiving SCI.

They were given meloxicam (5 mg/kg, subcutaneously [SC]) and lactated Ringer’s solution (0.5 mL, intraperitoneally) immediately after surgery, and left to recover on a heated pad. The mice were also administered 0.9% sterile saline daily (0.5 mL) for the first 48 h after surgery, to maintain hydration. Subsequent administration of saline was given only as needed. Mice received the antibiotic Baytril (2.5 mg/kg, SC) immediately after surgery and daily each morning up to 7 days post-operation (dpo) to minimize the risk of urinary tract or bladder infection in SCI animals. Experimenters manually expressed SCI mice bladders twice daily for the duration of experiments. Mice were assessed for impairment of locomotor function at 1 dpo using the Basso Mouse Scale (BMS) [22], to ensure effectiveness of the injury. SCI mice were only included in the study if they recorded BMS scores of 0 or 1 at 1 dpo.

### Terminal Tissue Collection

At 1 day or 14 days after sham or SCI surgery, the mice were deeply anesthetized with isoflurane and oxygen, as described above. The kidneys and 1 cm of spinal cord encompassing the lesioned site were rapidly extracted and flash-frozen in liquid nitrogen for subsequent cellular assays. The lesioned spinal cord and 1 kidney per subject were processed for the extraction of both total RNA (RNeasy Mini Kit; Qiagen, Valencia, CA) and total protein (see below) using procedures we routinely use [8, 9, 31].

### qRT-PCR and mRNA Quantification

Total RNA (100 ng) was converted into cDNA using TaqMan EZ RT-PCR Core reagents (Applied Biosystems, Carlsbad, CA) and the mRNA levels of *C-fos*, and the pro-inflammatory cytokines (*Tnfα*, *Il-1β*, and *Il-6*) were measured by TaqMan quantitative real-time (qRT)-PCR using a 7900HT Fast Real-Time PCR System (Applied Biosystems, Carlsbad, CA), as replicates. *β-actin* served as a control gene. The probes, forward and reverse primers for the targets [*C-fos* (Mm00487425_m1), *Il-1β* (Mm00434228_m1), *Il-6* (Mm00446190_m1), *Tnfα* (Mm00443258_m1), and *β-Act* (Mm02619580_g1)] were obtained from ThermoFisher Scientific, Waltham, MA. The 2 − ^ΔΔCT^ method was used to analyze the relative changes in gene expression. The mRNA expression for each gene of interest was normalized to *β-actin* expression and presented as a fold change increase or decrease in SCI and sham experimental groups compared to the naïve controls, which were normalized to 1.

### Western Blot and Protein Quantification

Western blotting was used for quantification of the protein expression of C-fos and TNFα in renal tissue and the lesioned spinal cord. Western blotting was also done for IL-1β and IL-6 protein, but their expressions were not reliably observed in renal tissue, unlike their expression in all spinal cord tissue assayed. Therefore, we limited our protein assessment by western blot to C-fos and TNFα. Glial fibrillary acidic protein (GFAP) expression in the lesioned spinal cord only was also assessed to further demonstrate that both T4 and T10 SCI similarly increased GFAP expression in the damaged spinal cord. Following RNA extraction, total protein was extracted from the remaining organic layer using QIAzol™ lysis reagent (Qiagen, Valencia, CA), as we previously reported [8, 9, 31]. Equal amounts (40 μg) of total protein were subjected to SDS-PAGE. Following transfer onto PVDF membranes (Millipore, Bedford, MA), the blots were blocked for one hour in 5% blotting grade milk (BioRad, Hercules, CA) in Tris-buffered saline Tween-20 (TBST). After blocking, the blots were incubated overnight at 4° C in primary antibody diluted in blocking solution as follows: C-fos (1:250; #SC-52, Santa Cruz Biotech, Dallas, TX), TNFα (1:500; #NB600-587, Novus Biological, Littleton, CO), GFAP (1:5000; #NB300-141, Novus Biological, Littleton, CO), and β-tubulin (1:1000; #05–661, Upstate Cell Signaling, Lake Placid, NY) served as a control. The following day, blots were washed in TBST (3 × 10 min) at room temperature then incubated in horseradish peroxidase-conjugated goat anti-rabbit or anti-mouse secondary antibodies (1:5000; #31,460 or 31,430, respectively; Pierce, Rockford, IL) for 1 h at room temperature. Following another 3 × 10 min washes, the blots were developed with standard enhanced chemiluminescence and imaged with Azure Biosystems c400 Western Blot Imaging System. Ratios of the integrated densitometry of each protein of interest to the loading control (β-tubulin) were calculated with AlphaView Software by ProteinSimple, normalized to naïve controls and averaged for animals within each group. The data are presented as a fold change in experimental groups (SCI and sham) compared to naïve control (1).

### Histological Verification of SCI

Mice were sacrificed one day after T4 and T10 SCI (*n* = 2 each) for histological assessment of the lesioned spinal cord. Luxol fast blue and hematoxylin and eosin staining methodologies which examine myelin and general structure of the injured spinal cord were undertaken as we previously described [32]. We also performed fluorescent histology to assess GFAP expression in the lesioned spinal cord dorsal horn 1 day after T4 and T10, using routinely used methodologies, e.g., [8]. For fluorescent histology, the spinal cord sections were blocked in 5% donkey serum, then incubated in GFAP antibody raised in rabbit (1:1000, # NB300-141) overnight. They were then incubated in Cy3- conjugated donkey anti-rabbit secondary antibody (Jackson ImmunoResearch Lab #711–165-152) for 1 h. Additional details on these methodologies and the results are provided as supplemental material.

### Statistics

All statistical analyses and correlations were undertaken with GraphPad Prism v9. One-way ANOVA with the recommended Tukey’s multiple comparison tests was used to analyze differences in gene/protein expression among the individual experimental groups (naïve, sham, and SCI) in each cohort. These analyses were undertaken to examine the effect SCI has on the expression levels of the inflammatory mediators at both acute and sub-chronic time point, after T4 and T10 SCI. Hence, the current study did not make comparisons between the different timepoints (1 day versus 14 days) or injury levels (T4 versus T10). Pearson correlation calculations and simple linear regression were also undertaken to examine correlations of the various cytokines and C-fos expression levels in the kidney and lesioned spinal cord, and also relationships between the mRNA and protein expression levels of C-fos and TNFα in the kidney and spinal cord. In text and figures, all data are presented as mean ± SEM. In the figures, *p < 0.05, **p < 0.01, ***p < 0.001, and ****p < 0.0001 show significance compared to naïve and/or sham.

## RESULTS

This study investigated the effect of SCI on the expression profile of inflammatory markers in the kidney. To further examine whether SCI indiscriminately induces renal inflammation, we assessed the relative abundance of the various inflammatory mediators at 1 day and 14 days after a high (T4) and low (T10) contusion SCI. We also assessed changes in the lesioned spinal cord. As also shown in Supplemental Fig. 1, SCI at both T4 and T10 produced noticeable damage to the spinal cord (Fig. S1A), and increased GFAP expression at 1 day (Fig. S1B) and at 14 days (Fig. S1C) after SCI. Each experimental cohort consisted of 3 experimental groups: naïve (*n* = 6), sham (*n* = 5), and SCI (*n* = 7–8). A total of 73 (37 female and 36 male) mice were used in the study as follows: T10, 1 day (10 female, 9 male), T10 14 days (8 female, 10 male), T4, 1 day (8 female, 10 male), and T4, 14 days (11 female, 7 male). There were no notable sex differences, so the results from female and male mice are combined. Also, each treatment group had an appropriate number of mice of each sex.

**Fig. 1:**
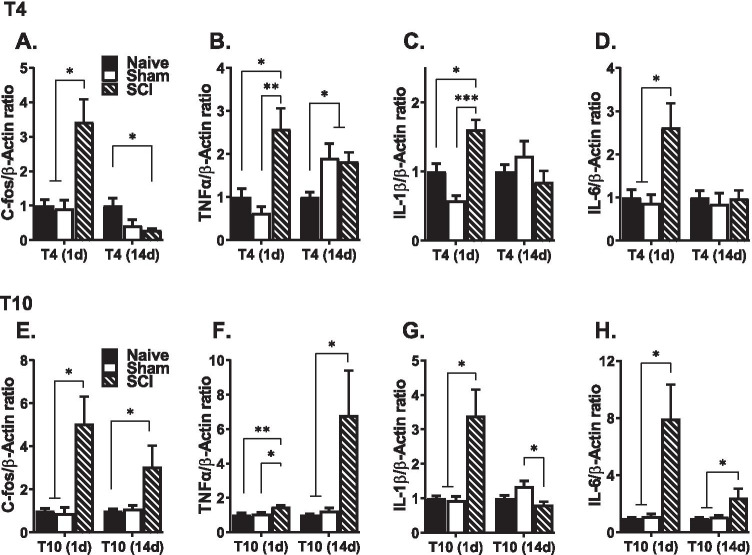
Changes in mRNA expression levels in renal tissue. **A**–**D** T4 SCI increased the mRNA expression levels of *C-fos*, *Tnfα*, *Il-1β*, and *Il-6*, in the kidney, 1 day after injury. At 14 days, *C-fos* mRNA level in the kidney was decreased in the SCI group. Meanwhile, *Tnfα* mRNA was increased in the kidney of sham and SCI mice compared to naïve controls. **E**–**H** *C-fos*, *Tnfα*, *Il-1β*, and *Il-6* mRNA levels were significantly elevated in kidney tissue, 1 day after T10 SCI. *C-fos*, *Tnfα*, and *Il-6* remained elevated at 14 days after T10 SCI, while IL-1β was reduced (**G**) (*p < .05, **p < .01, and ***p < .001 compared to naïve and/or sham subjects).

### Changes in mRNA Expression

**Fig. 2:**
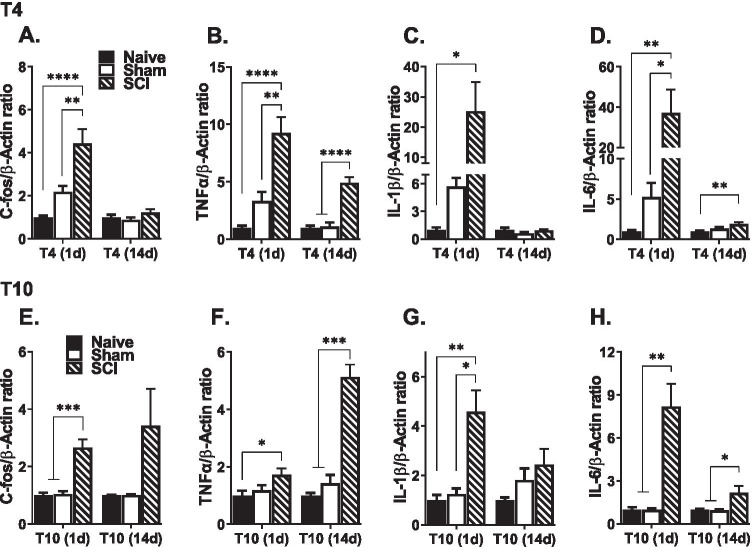
Changes in mRNA expression levels in lesioned spinal cord. **A**–**D** T4 SCI robustly increased the mRNA expression levels of *C-fos*, *Tnfα*, *Il-1β*, and *Il-6*, in the spinal cord epicenter, 1 day after injury. At 14 days, the mRNA expression levels of *Tnfα* and *Il-6* remained elevated. **E**–**H** After T10 SCI, *C-fos*, *Tnfα*, *Il-1β*, and *Il-6* mRNA levels were significantly elevated in spinal cord at 1 day. Spinal expression levels of *Tnfα* and *Il-6* remained increased at 14 days after T10 SCI, although *C-fos* and *Il-1β* were unchanged (*p < .05, **p < .01, and ***p < .001 compared to naïve and/or sham subjects).

We assessed the expression levels of *C-fos*, *Tnfα*, *Il-1β*, and *Il-6* mRNA levels in the kidney (Fig. 1) and spinal cord (Fig. 2) at 1 day and 14 days after T4 (A–D) and T10 (E–H) contusion injury. One-way ANOVA analyses were performed to analyze group differences among the three treatment groups (naïve, sham, and SCI) in each of the 4 cohorts.

### Effect of SCI on Renal C-fos and Pro-inflammatory Cytokine Levels

Renal *C-fos* mRNA was increased 1 day after T4 injury (F _(2, 13)_ = 7.8, *p* = 0.006) but was downregulated at 14 days (Fig. 1A). Specifically, *C-fos* was decreased in SCI mice (0.30 ± 0.05) compared to naïve control (*F*_(2, 15)_ = 5.9, *p* = 0.013). However, following T10 SCI, *C-fos* mRNA expression was significantly increased in the kidney of SCI subjects at 1 day (*F*_(2, 18)_ = 5.5, *p* = 0.014) and 14 days (*F*_(2, 13)_ = 4.6, *p* = 0.032), compared to both naïve and sham groups (Fig. 1E).

The mRNA expression levels of *Tnfα* (Fig. 1B, F), *Il-1β* (Fig. 1C, G), and *Il-6* (Fig. 1D, H) were also assessed. Significant group differences in *Tnfα* expression were observed at both 1 day (*F*_(2, 13)_ = 7.8, *p* = 0.006) and 14 days (*F*_(2, 15)_ = 5.0, *p* = 0.022) in the T4 groups. Notably, *Tnfα* mRNA expression was increased in the kidney of both SCI (1.8 ± 0.2) and sham (1.9 ± 0.3) subjects compared to naïve control mice 14 days after T4 injury (Fig. 1B). *Tnfα* was also significantly increased in the kidney at 1 and 14 days after T10 SCI (*F*_(2, 18)_ = 7.3, *p* = 0.005, and *F*_(2, 13)_ = 5.3, *p* = 0.021, respectively), compared to both naïve and sham controls (Fig. 1F).

*Il-1β* mRNA expression was increased in the kidney 1 day after T4 SCI (*F*_(2, 11)_ = 18.0, *p* = 0.0003) and T10 SCI (*F*_(2, 17)_ = 9.2, *p* = 0.002) (Fig. 1C, G), and *Il-6* mRNA was similarly increased in the kidney 1 day after both T4 (*F*_(2, 11)_ = 6.9, *p* = 0.012) and T10 SCI (*F*_(2, 17)_ = 11.8, *p* = 0.001) (Fig. 1D, H), compared to sham and naïve. Interestingly, at 14 days post-T10 SCI, renal *Il-1β* expression was decreased in SCI subjects (0.8 ± 0.1) compared to sham control (1.3 ± 0.2), but unchanged compared to naïve control (*F*_(2, 14)_ = 5.3, *p* = 0.019) (Fig. 1G). Also, at 14 days after T10 SCI, *Il-6* was increased in SCI subjects (*F*_(2, 13)_ = 5.2, *p* = 0.022) (Fig. 1H). No changes were observed in renal *Il-1β* and *Il-6* mRNA expression 14 days after T4 SCI. Altogether, these results show that systemic inflammation extends to the kidney after both high and low thoracic spinal cord injury.

### Effect of SCI on Spinal C-fos and Pro-inflammatory Cytokine Levels

As shown in Fig. 2A and E, *C-fos* mRNA was robustly increased in the lesioned spinal cord 1 day after both T4 (*F*_(2, 12)_ = 23.9, *p* < 0.0001) and T10 (*F*_(2, 16)_ = 18.6, *p* < 0.0001) injuries. No differences in expression were observed at 14 days after either injury. All three cytokines were significantly increased in the lesioned spinal cord after T4 and T10 injury (Fig. 2). Specifically, *Tnfα* mRNA levels were greatly increased 1 and 14 days after T4 (*F*_(2, 13)_ = 18.7, *p* = 0.0001, and *F*_(2, 14)_ = 30.9, *p* < 0.0001, respectively) and T10 (*F*_(2, 13)_ = 3.9, *p* = 0.046, and *F*_(2, 15)_ = 55.5, *p* < 0.0001, respectively) injuries (Fig. 2B, F). Likewise, *Il-1β* mRNA was increased in the spinal cord although only at 1 day after T4 (*F*_(2, 10)_ = 6.2, *p* = 0.018) and T10 (*F*_(2, 17)_ = 9.2, *p* = 0.002) injuries (Fig. 2C, G). Unlike *Il-1β*, *Il-6* expression level was increased in the spinal cord of SCI subjects at 1 and 14 days after T4 (*F*_(2, 10)_ = 9.9, *p* = 0.004, and *F*_(2, 15)_ = 6.4, *p* = 0.001, respectively) and T10 (*F*_(2, 17)_ = 11.8, *p* = 0.001, and *F*_(2, 15)_ = 4.8, *p* = 0.025, respectively) injuries (Fig. 2D, H). Overall, these results reveal a robust increase in inflammatory mediators in the lesioned spinal cord after both T4 and T10 SCI.

### Changes in Protein Expression

**Fig. 3:**
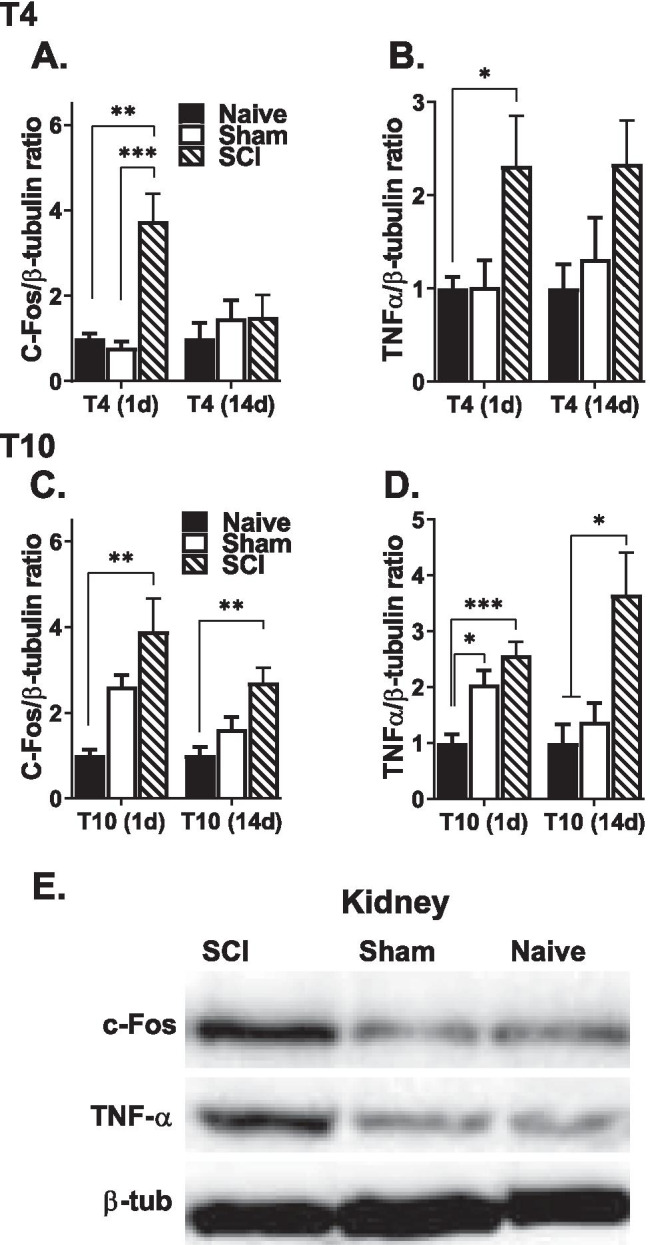
Changes in C-Fos and TNFα protein expressions in the kidney. T4 SCI increased C-fos (**A**) and TNFα (**B**) protein expression in the kidney, only at 1 day after SCI. The protein levels of C-fos (**C**) and TNFα (**D**) were significantly upregulated in the kidney 1 and 14 days after T10 SCI compared to naïve and/or sham groups. **E** Representative images of C-Fos, TNFα, and β-tubulin (loading control) western blot from T10, 1-day cohorts are shown (*p < .05, **p < .01, and ***p < .001 compared to naïve and/or sham subjects).

**Fig. 4:**
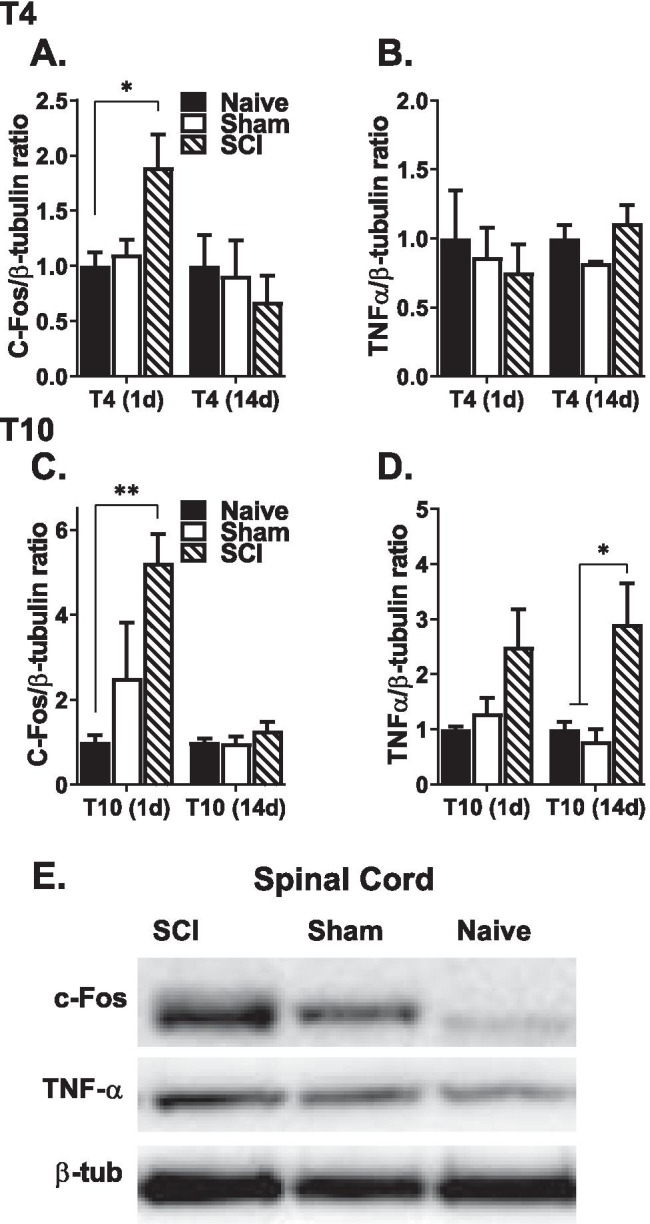
Changes in C-fos and TNFα protein expressions in the lesioned spinal cord. Compared to naïve only, C-fos protein is increased in the spinal cord of SCI mice 1 day after T4 SCI (**A**) and T10 SCI (**C**). Whereas TNFα protein was unchanged after T4 SCI (**B**), it was increased in the lesioned spinal cord, 14 days after T10 SCI (**D**). **E** Representative images of C-fos, TNFα, and β-tubulin (loading control) western blot in the spinal cord from T10, 1-day cohorts are shown (*p < .05 and **p < .01 compared to naïve and/or sham subjects).

We also used western blot to measure the protein expression levels of C-fos and TNFα in the kidney and spinal cord. Although the TNFα antibody recognizes both soluble (17 kD) and cell-bound (26 kD) TNFα, the 26 kD precursor protein was more reliably observed in all tissue and hence reported here. These results depicting changes in C-Fos and TNFα protein in the kidney and spinal cord after T4 (top) and T10 (bottom) injury are shown in Figs. 3 and 4.

### SCI Increased C-fos and TNFα Protein Levels in the Kidney

Significant group differences in renal C-fos protein expression levels were observed 1 day after both T4 (*F*_(2, 14)_ = 16.4, *p* = 0.0002) and T10 injury (*F*_(2, 15)_ = 7.7, *p* = 0.005). There were no group differences in C-fos protein expression in the kidney 14 days after T4 injury, although it remained elevated 14 days after T10 SCI [2.7 ± 0.4 (*F*_(2, 12)_ = 7.0, *p* = 0.009)] (Fig. 3A, C). TNFα protein levels were also increased by SCI (Fig. 3B, D). Specifically, there was a significant increase in TNFα protein 1 day after T4 SCI (*F*_(2, 13)_ = 4.158, *p* = 0.040). A similar group effect was observed in the T10 SCI group 1 day after injury (*F*_(2, 16)_ = 12.8, *p* = 0.001), although both sham (2.0 ± 0.3) and SCI (2.6 ± 0.2) groups had increased levels of TNFα in the kidney. TNFα protein remained increased at 14 days only in the SCI group (*F*_(2, 11)_ = 7.1, *p* = 0.010).

### SCI Increased C-fos and TNFα Protein Levels in the Lesioned Spinal Cord

While C-fos protein was elevated in the lesioned spinal cord of SCI mice 1 day after T4 (*F*_(2, 12)_ = 4.8, *p* = 0.030) and T10 injury (*F*_(2, 12)_ = 7.4, *p* = . 008), compared only to naïve control, its expression was unchanged 14 days after either T4 or T10 SCI (Fig. 4A, C). Overall, these changes in C-fos protein level are consistent with that seen in *C-fos* mRNA levels, in that while changes in the spinal cord were only observed 1 day after injury, renal C-fos expression remained elevated up to 14 days after T10 SCI. Unlike the robust effects seen in the kidney, there were no changes in TNFα protein expression in the spinal cord 1 day or 14 days after T4 SCI, or 1 day after T10 SCI. However, there was a significant group effect observed at 14 days after T10 injury (*F*_(2, 10)_ = 7.4, *p* = 0.011) (Fig. 4B, D).

### Correlational Analyses

**Fig. 5:**
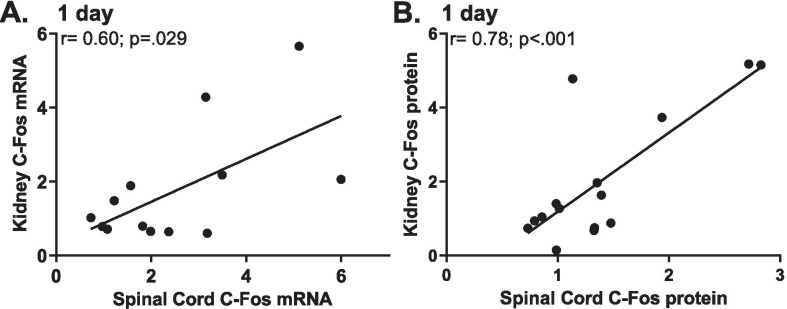
Correlations between renal and spinal mRNA and protein expression after T4 SCI. At 1 day after T4 SCI, *C-fos* mRNA (**A**) and protein (**B**) levels in the kidney and spinal cord were strongly correlated.

**Fig. 6:**
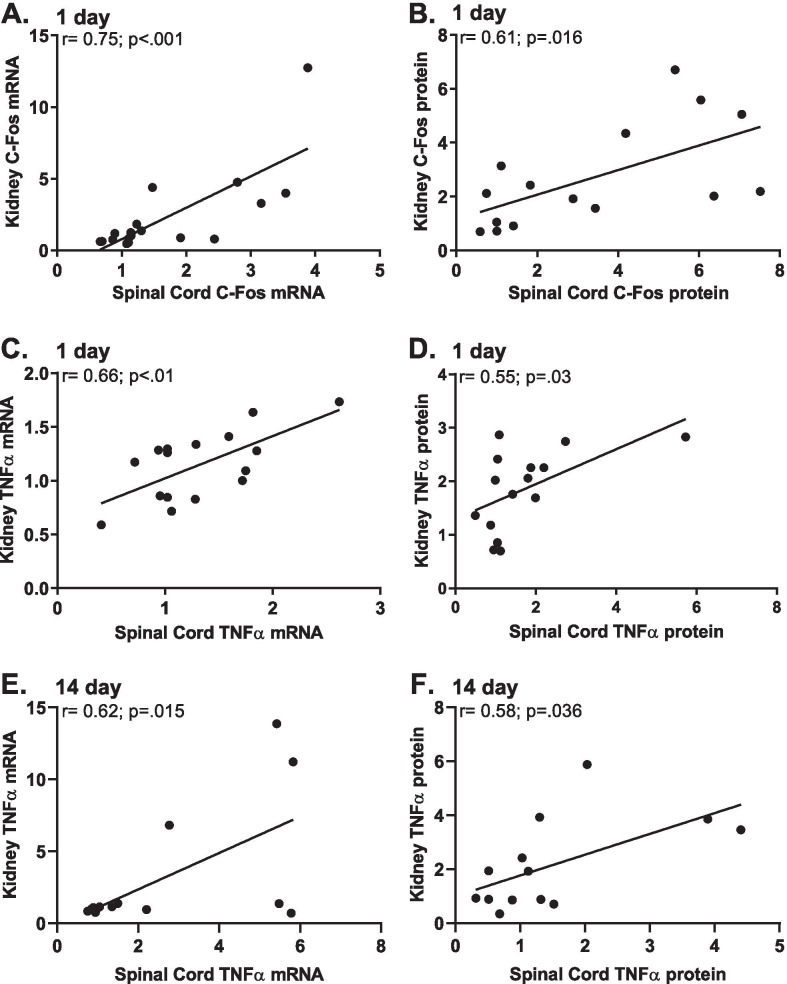
Correlations between renal and spinal mRNA and protein expression after T10 SCI. At 1 day after T10 SCI, strong correlations between spinal and renal *C-fos* mRNA (**A**), C-fos protein (**B**), *Tnfα* mRNA (**C**), and TNFα protein (**D**) expressions were observed. Also, in the 14-day group, there was a strong correlation between TNFα mRNA (**E**) and protein levels (**F**) in the kidney and spinal cord.

Additional analyses were conducted to examine correlations between the spinal and renal expression levels of the various inflammatory mediators (Figs. 5 and 6), and between the mRNA and protein expressions of C-fos and TNFα (Table 1). For T4 subjects, assessed at 1 day, we observed strong correlations between kidney and spinal cord *C-fos* mRNA (Fig. 5A) and C-fos protein (Fig. 5B) expression. No such correlations were observed at 14 days. Similar correlations were observed 1 day and 14 days after T10 injury. Specifically, as shown in Fig. 6, there were strong correlations between kidney and spinal cord C-fos mRNA (Fig. 6A) and protein (Fig. 6B) expression levels, and between TNFα mRNA (Fig. 6C) and protein (Fig. 6D) in the 1-day group. Also, TNFα mRNA (Fig. 6E*)* and protein (Fig. 6F) levels in the kidney and spinal cord were strongly correlated in the T10, 14-day group. These strong correlations between renal and spinal expressions of mRNA and protein support the notion that SCI, regardless of the injury level, produces a systemic inflammatory response evidenced in part by robust increases in inflammatory mediators both centrally (lesioned spinal cord) and in the periphery (kidney).

**Table 1:** Correlation of C-fos and TNF mRNA and protein levels in the kidney and spinal cord. All *r* and *p* values are provided, with statistical significance denoted by asterisks (*p < .05, **p < .01, and ***p < .001))

		T4 (1 day)	T4 (14 days)	T10 (1 day)	T10 (14 days)
C-FOS	Kidney	***r* = 0.67** *p* = .007**	*r* = − 0.05 *p* = .837	***r* = 0.80** *p* = .0001***	*r* = 0.39 *p* = .192
	Spinal cord	***r* = 0.72** *p* = .006**	*r* = − 0.42 *p* = .085	***r* = 0.67** *p* = .012*	*r* = − 0.10 *p* = .734
TNFα	Kidney	*r* = 0.42 *p* = .131	*r* = 0.13 *p* = .667	*r* = 0.37 *p* = .131	***r* = 0.73** *p* = .007**
	Spinal cord	*r* = − 0.34 *p* = .218	*r* = 0.22 *p* = .456	*r* = 0.57 *p* = .088	***r* = 0.76** *p* = .004**

We also examined the relations between the mRNA and protein levels of C-fos and TNFα in the kidney and spinal cord in all 4 groups (Table 1). C-fos mRNA and protein expression levels were positively correlated in renal and spinal tissue at 1 day only in both T4 and T10 injury groups. On the other hand, TNFα mRNA and protein expression were correlated in both renal and spinal tissue only in the T10 (14 day) group.

## DISCUSSION

The primary objective of the study was to assess the effect of SCI on the expression profiles of several pro-inflammatory mediators in the kidney and lesioned spinal cord. We examined temporal and spatial-related changes in the expression of C-fos, TNFα, IL-1β, and IL-6 after high and low thoracic level SCI, and observed an overwhelming increase in the expression levels of these inflammatory mediators at both locales. Interestingly, regardless of the location of the injury, all 4 targets were significantly upregulated in the kidney and spinal cord of SCI subjects at the acute timepoint, compared to control subjects. This observation clearly demonstrated that SCI produces a robust systemic inflammatory response. The systemic nature of SCI, at least during the acute stages of injury, is further supported by the correlation analyses, which generally showed strong positive correlations between the renal and spinal expression levels of both C-fos and TNFα 1 day after both T4 and T10 SCI.

Two weeks after SCI, some moderate differences in the expression profiles of the targets emerged. Specifically, *C-fos*, *Il-6*, and *Tnfα* mRNA remained elevated in the kidney after T10 SCI, as did C-fos and TNFα protein. In contrast, in the T4 SCI group, only *Tnfα* mRNA and protein were increased in the kidney. Although a direct comparison of the efficacy of T4 versus T10 SCI on the expression levels of the cellular targets was not undertaken, these observations seem to suggest that T10 SCI had a more lasting effect on cytokines and C-fos levels in renal tissue. In the spinal cord, similar increases in *Il-6* and *Tnfα* mRNA expression were seen after both high and low thoracic SCI, although only in the T10 group did TNFα protein remain elevated to 14 days. Thus, it appears that there is a spatial component to the SCI-induced inflammation at peripheral targets, even though widespread increases in inflammatory mediators were seen 1 day after injury regardless of the injury level.

A key component of any systemic inflammatory response involves the vascular system. An increased circulation of immune cells and proinflammatory mediators contributes to the influx of inflammatory cells into peripheral organs, including the kidney, in diseased conditions (see review by Bonavia and Singbart [33]). In addition to changes in blood flow that contribute to the renal inflammatory response, compromised neural innervation of the kidney after SCI may be similarly implicated in the maladaptive cellular plasticity, and consequently renal dysfunction. The kidney is innervated by both afferent and efferent nerves [34, 35]. The “efferent” preganglionic sympathetic nervous innervation to the kidney, arises mainly from the intermediolateral column of the spinal cord at T8 to lumbar 1 segments. Meanwhile, the thoracic splanchnic nerves provide afferent signaling from the kidney to the central nervous system (CNS). Renal afferent nerve fibers consist of pain fibers (C and Aδ-fibers) and mechanoreceptors. In the CNS, injury-induced activation of pain fibers has been shown to increase C-fos and cytokines expression [8, 31]. Therefore, it is conceivable that abnormal neural activity in the kidney plays a role in exacerbating the renal inflammatory response after SCI. We were not able to delineate whether an injury at T10 has a more direct effect on renal blood flow and neural innervation than an injury at T4; the consequence of which could be a longer-lasting inflammatory response. However, it should be noted that the critical outcome of this study is that SCIs above or at the segmental level of the kidney produced an acute, albeit robust, systemic inflammatory response, evidenced as an increase in inflammatory mediators in the kidney. Furthermore, injuries at both levels resulted in similar cellular and morphological plasticity centrally.

Acute inflammation in the kidney after high thoracic level spinal cord injuries has been previously demonstrated. For instance, Gris et al. [10] showed that there is an invasion of neutrophils into the kidney acutely after T4 SCI, and Bao et al. [36] reported more robust renal inflammation at an acute timepoint following T4 SCI than after T12 SCI in adult rats. However, Hubscher et al. [37] reported elevated levels of cluster of differentiation molecule 11b (CD11b), an adhesion molecule known to promote cell–cell adhesion in inflammation in the kidney of adult rats approximately 12 weeks after T9 contusion SCI. Together, these observations align with our finding that there is widespread increase in inflammatory cytokines in renal tissue at acute timepoints following SCI, regardless of the injury level, although by 14 days, there appears to be a spatial preference where injury at T10 has a greater effect than T4. They also demonstrate that SCI exerts systemic effects marked by, among other consequences, an inflammatory response that extends to distal organs.

Downstream cascades activated by the inflammatory mediators IL-1β, IL-6, and TNFα can have several consequences on renal pathophysiology and dysfunction. For example, TNFα has been linked to apoptosis and related cell death within the injured spinal cord [8, 38, 39], and in renal tissue, it is associated with renal failure [40, 41]. It can be assumed that similar cell death mechanisms will be initiated in the kidney as a consequence of SCI-induced inflammation. TNFα and IL-6 have also been shown to contribute to blood pressure alterations by promoting vascular and renal damage [42, 43]. There is extensive evidence that both hypertension and hypotension, common consequences of SCI, can be associated with inflammation. Altogether, these fore-mentioned studies suggest that systemic inflammation extending to the kidney could exert negative consequences on the renal system. These may include, although not limited to, the exacerbation of kidney disease and promotion of blood pressure dysregulation. As was previously noted, C-fos, like the inflammatory cytokines, is increased in the kidney in diseased or injured conditions [14–17], an observation also seen here. It was previously shown that the selective C-fos inhibitor, T-5224, reduced lipopolysaccharide-induced increases in serum TNFα levels, and importantly reduced intercellular adhesion molecule -1 expression and morphological evidence of tubular injury in the kidney [16], thereby providing additional evidence that C-fos contributes to kidney pathophysiology following systemic inflammation. In the CNS, C-fos is typically associated with increased neural activity and nociception [44, 45], including after SCI [46]. We have previously shown that C-fos expression is increased in the lesioned thoracic and spared lumbar spinal cord after contusion SCI, with greater increases after concurrent noxious stimulation [8, 9]. In peripheral cells, C-fos expression is associated with programmed cell death and tissue remodeling [47, 48]. Hence, it is likely that increased levels of C-fos and the inflammatory cytokines substantially intensify renal damage after SCI by initiating cell death mechanisms. The increased expression of C-fos in the kidney further suggests that systemic inflammation also engages cyclic adenosine monophosphate/calcium-dependent and kinase pathways which are known to rapidly activate C-fos. Future studies will focus on elucidating the mechanisms by which C-fos leads to renal damage after SCI.

Immune cells are an important source of inflammatory cytokines. It will be critical to know whether immune cells are differentially trafficked to or expressed in the kidney and spinal cord immediately or at 2 weeks post-SCI. As mentioned above, SCI can cause an increase in immune cells and inflammatory mediators in the circulation. Both microglia and macrophages are critical mediators of the secondary effects that occur in the injured spinal cord (see reviews [49, 50]). Monocyte-derived macrophages are implicated in kidney disease triggered by inflammation (see reviews [51, 52]). They are trafficked to the inflamed kidney following ischemia–reperfusion injury [53] or puromycin-induced renal disease [54], via chemokine-dependent mechanisms. In addition to the trafficking of blood-derived immune cells, renal endothelial, and epithelial cells can themselves release pro-inflammatory cytokines in situ in response to injury [55, 56]. Clearly, a myriad of cellular mechanisms interplay in the inflammatory processes that occur in the kidney after SCI. However, subsequent studies will examine the involvement of monocyte-derived macrophages and other immune cells such as neutrophils in the renal inflammatory response triggered by SCI. While this study did not assess renal function or subsequent behavioral consequences, we postulate that SCI induces robust systemic ‘renal’ inflammation that can severely impair the cellular integrity of kidney tissue, promote renal dysfunction and consequently, lead to blood pressure dysregulation after SCI. We further propose that after SCI, immune cells in the circulation play a pivotal role in the inflammatory response observed in the kidney, at least acutely. Notably, in this study, we showed for the first time that SCI indiscriminately leads to an upregulation of inflammatory mediators that extends beyond the injured spinal cord to the kidney shortly after SCI.

SCI has been shown to induce systemic inflammatory response syndrome (SIRS) [36, 57] which is defined as the body’s response to an infection or non-infectious insult. SIRS that typically accompanies disease states such as sepsis is implicated in multiple organ dysfunction (e.g., [58]). As it relates to renal function, several studies have shown that SIRS and accompanying sepsis promote acute kidney disease (see reviews [59–61]). The results of the current study supported the notion that the acute inflammatory response induced by thoracic level SCI can similarly exacerbate kidney disease, which could eventually lead to renal failure. Importantly, it was previously shown that SCI also induces septicemia [62, 63]. Overall, this study highlighted that systemic inflammation is indeed a noteworthy effect of SCI that has the potential to induce widespread organ dysfunction.

## CONCLUSION

Recent studies have indicated that SCI exerts systemic effect, including an inflammatory response that spreads to peripheral tissue. In this study, we assessed changes in the expression levels of key pro-inflammatory mediators in the kidney after T4 and T10 contusion SCI. We showed that whereas both injuries induced robust renal inflammation shortly after SCI, more sustained effects were seen after T10 SCI. The results of the study demonstrated that SCI exerts far-reaching consequences and also suggested that peripheral organ function can be impaired by inflammatory mediators after SCI. Future studies will explore the effect systemic inflammation has on renal tissue morphology and renal function after SCI.

## SUPPLEMENTARY INFORMATION

Below is the link to the electronic supplementary material.

Supplementary file1 (DOCX 20 KB)

## Data Availability

The data used to support the findings of this study are included within the article.
