# Supplementary material for: Spinal Cord Injury Increases Pro-inflammatory Cytokine Expression in Kidney at Acute and Sub-chronic Stages
Source: Inflammation. 2021 Aug 21;44(6):2346–61. doi: 10.1007/s10753-021-01507-x (PMC8616867; doi:10.1007/s10753-021-01507-x)
Supplement: Supplementary file 1 — Supplementary file1 (DOCX 20 KB) [file 10753_2021_1507_MOESM1_ESM.docx]

**TITLE:** Spinal Cord Injury increases pro-inflammatory cytokine expression in kidney at acute and sub-chronic stages.

**Authors:**

Shangrila Parvin^1^, Clintoria R. Williams^1,2^, Simone A. Jarrett^1^ and Sandra M. Garraway^1^

^1^ Department of Physiology, Emory University School of Medicine, Atlanta, GA.

^2^ Neuroscience, Cell Biology and Physiology, Wright State University, Dayton, OH.

**Supplementary Material**

**Methods:**

Briefly, mice were transcardially perfused with phosphate-buffered saline (PBS) followed by 4% paraformaldehyde (PFA) in PBS. The spinal cord, including the lesioned epicenter, was dissected, post-fixed in 4% PFA, then transferred to 30% sucrose for cryoprotection, and subsequently cut at 20 μm transverse or longitudinal sections with a cryostat (Leica Microsystems, Buffalo Grove, IL). The slide-mounted spinal cord sections were used for Luxol Fast-blue, hematoxylin and eosin stained, and cresyl violet for cytoarchitectural evaluation (Nissl substances). For GFAP fluorescent histology, the slides were washed in 1 X phosphate buffered saline (PBS) with 0.1% Triton X-100. Following secondary antibody incubation, the slides were washed 3 times in 1 X PBS-T. the slides were mounted in Prolong Gold anti-fading mounting medium (Invitrogen, Eugene, OR) and coverslipped.

**Results:**

**Figure S1:** [**A**] H & E histological staining showed spinal cord lesion zones at 1 day after T4 and T10 SCI. [**B**] GFAP immunolabeling seen in the dorsal horn of the spinal cord 1 day after T4 and T10 SCI. [**C**] Western blotting showed a significant increase in GFAP expression in the lesioned spinal cord 14 days after (**ii**)T4 [F _(2, 10)_ = 10.8, P = .003] and (**iv**) T10 [F _(2, 12)_ = 29.8, P < .0001] SCI. There were no significant increases in GFAP expression 1 day after either SCI (**i** and **iii**).
